# Dimensions of corvid consciousness

**DOI:** 10.1007/s10071-025-01949-y

**Published:** 2025-05-02

**Authors:** Walter Veit, Heather Browning, Elias Garcia-Pelegrin, James R. Davies, Jamie G. DuBois, Nicola S. Clayton

**Affiliations:** 1 University of Reading, Reading, UK; 2 University of Southampton, Southampton, UK; 3 National University of Singapore, Singapore, Singapore; 4 University of Cambridge, Cambridge, UK; 5 University of Bristol, Bristol, UK

**Keywords:** Animal consciousness, Dimensions of consciousness, Bird consciousness, Corvids, Animal cognition

## Abstract

Corvids have long been a target of public fascination and of scientific attention, particularly in the study of animal minds. Using Birch et al.’s (2020) 5-dimensional framework for animal consciousness we ask what it is like to be a corvid and propose a speculative but empirically informed answer. We go on to suggest future directions for research on corvid consciousness and how it can inform ethical treatment and animal welfare legislation.

## Introduction

Within the last few decades there has been a significant shift in our understanding of the intelligence of birds. Whereas derogatory words like ‘birdbrain’ were once used, the scientific community as well as the lay public are now recognizing how smart many birds are. This is especially true for the corvids, such as crows and jays, whose learning and problem-solving abilities rival even those of the great apes (Emery and Clayton 2004; van Horik et al. 2012; Kabadayi et al. 2016), earning them the moniker ‘feathered apes’ (Emery 1998; Clayton and Emery 2004; Clayton 2012; Güntürkün et al. 2017). With ongoing research and developments in the science of corvid brains and behaviour, we can finally make progress on the perennial question (first raised by Nagel 1974): what is it *like* to be a bird, and, in particular, a corvid? A question that was once thought to be beyond the scope of science has, over the last decade, become a legitimate question to ask.

The last decade has seen the growth of the science of animal consciousness, with the ‘Cambridge Declaration on Consciousness’ in 2012 and the founding of *Animal Sentience*, the first journal dedicated to animal consciousness in 2015. Although there had been earlier attempts to create a science of animal consciousness—most prominently a call made to establish a ‘cognitive ethology’, by the American ethologist (and discoverer of bat echolocation) Donald Griffin in his 1976 book ‘The Question of Animal Awareness’—the behaviourist spirit continued for some to deter scientists from attempting such a project (Allen and Trestman 2017; Blumberg and Wasserman 1995). With more recent shifts in the field, we are now approaching a consensus that all mammals and birds are conscious (Low et al. 2012; Andrews et al. 2024), rather than just humans, thus shifting the field away from its preoccupation with the question of *which* animals are conscious and more towards a focus on the levels of consciousness, different modalities of consciousness, and what the subjective experiences of different animals consist of. Although there are many animal groups about which there is still controversy regarding their sentience (e.g. invertebrates), there are now serious efforts to understand the contents of animal experience.

It is within this context that Birch et al. (2020), have suggested that a comparative study of animal consciousness should recognize five different dimensions of animal consciousness, each with different levels of richness, by creating hypothetical, but nevertheless conjectural, ‘consciousness profiles’ for nonhuman animals. These five dimensions are: sensory experience, evaluative experience, the experience of a self, and the unity of experience both immediate and across time calling the former unity and the latter temporality, though following Veit's (2023) amendment of this framework, we refer to these here as synchronic and diachronic unity. Here, we have made use of this five-dimensional framework to shape a review of the literature on corvids. It is not our goal here to make the case that corvids are conscious (i.e. have any type of subjective experience). Even discussions of human consciousness are unresolved in terms of what brain states are conscious, so the animal case is even more controversial. Instead, we simply assume the presence of consciousness in corvids as a starting point (in line with Andrews et al. 2024; Low et al. 2012) and aim to make progress on the question of *what it is like* to be a corvid if indeed they are conscious, as well as how to use this evidence to guide ethical and animal welfare considerations for corvids.

## Sensory richness in corvids

In their multi-dimensional approach to consciousness, Birch et al. define perceptual richness or what they call p-richness as the degree of detail in which an animal consciously perceives the world around them. As highlighted by the authors, the perceptual faculties of an animal vary depending on the sensory modalities; thus any measure of the perceptual experience of a species cannot be summarized by an overall level, but ought to be considered instead relative to the degree of richness in each sensorial modality. It is because of this that we prefer the term ‘sensory richness’. It is less evocative of vision and better reflects the multiple sensory modalities of animals. An obvious problem one immediately faces, however, is how to study the conscious perception of non-human animals without language?

Birch et al. suggest that a combination of neurological and behavioral experimentation might help. Nieder et al. (2020) investigated visual sensory consciousness by monitoring the responses of neurons in the pallial endbrain of two carrion crows (*Corvus corone corone*) by training them to report the presence or absence of a perceptually faint visual stimulus in a delayed detection task. The crows first viewed a stimulus presented near their threshold for perceptibility. This indicated that the crows only perceived the stimulus in reference to their own internal state. After the stimulus and a short delay, a cue was presented to indicate the motor action that the preceding stimulus required; this ensured that the crow could not prepare the response in advance. Nieder et al. report a two-stage process in the crow’s *nidopallium caudolaterale* (NCL). The first burst of activity is related to the unconscious detection of the visual stimulus. The second, delayed, burst of activity is related to the conscious perception of it. This is similar to the neuronal activity found in the primate cerebral cortex (Baars 2002; de Lafuente and Romo 2006; Edelman et al. 2005; Supér et al. 2001). This evidence suggests that this kind of sensory consciousness might have evolved in a common ancestor of both birds and mammals or that there might have been evolutionary convergence between corvids and primates (Nieder et al. 2020).

Despite some similarities in eye morphology with other vertebrates, such as humans, avian visual perception is characterized by acute vision and higher temporal resolution compared to most other vertebrates (Martin 2007; Butler et al. 2018). Temporal resolution, which refers to the amount of light information perceived at any given time, is an area where birds particularly excel (Bobrowicz and Osvath 2019). Birds must navigate a fast-paced world, dodging branches among treetops, foraging, or pursuing rapid and elusive prey, all while avoiding predation. This necessitates rich visual perception and the ability to process spatial information accurately while navigating at high speeds, a capability that has been strongly selected for in this taxon (Butler et al. 2018).

Flicker fusion refers to the threshold with which a flickering light is perceived as a continuous stream. The critical flicker fusion frequency (CFF) is the highest frequency at which light is still perceived as flickering (Rubene 2009). Behavioral experiments with passerines, including blue tits (*Cyanistes caeruleus*), collared flycatchers (*Ficedula albicollis*), and pied flycatchers (*Ficedula hypoleuca*), suggest that these birds can detect blinking light at frequencies of 129 Hz for blue tits, 127 Hz for collared flycatchers, and 137 Hz for pied flycatchers. They perceive flickering light as continuous at 131 Hz for blue tits, 141 Hz for collared flycatchers, and 146 Hz for pied flycatchers (Ikebuchi and Okanoya 1999; Boström et al. 2016). These frequencies are significantly higher—about 40 Hz more—than those of any other vertebrate, with humans averaging 60 Hz (Landis 1954; Shankar and Pesudovs 2007). Although CFF in corvids has not been extensively studied, Davidson et al. (2017) presented videos of moving objects to a sample of Eurasian jays (*Garrulus glandarius*) at a frame rate of 120 Hz. The jays had to choose the correct video to match a prior real-life display of these objects. Because this sample of jays demonstrated significant adeptness in making such comparisons between the real world and the videos displayed at a high temporal resolution, it is reasonable to assume that this species of corvid perceived the videos at 120 Hz as smooth motions rather than staggered flickering images. This flicker fusion frequency also informs the way a corvid might temporally perceive the world before interacting with it. Bobrowicz and Osvath (2019) used a forced-selection task to compare gaze durations in ravens (*Corvus corax*) and human participants as they assessed a selection of objects before making a choice. Their strong interest in interacting with objects (Jacobs et al., 2014) made it likely that they would pay close attention before making a selection. Similarly, humans were explicitly instructed to choose only one object, ensuring comparable engagement with the task. The study revealed that ravens’ gazes toward the objects lasted about half as long as those of humans. This difference may stem from the ravens' higher temporal resolution, suggesting that their faster perceptual processing is matched by a similarly rapid processing of the information they perceive.

Alongside their higher temporal resolution, most avian species, including corvids, have their eyes positioned laterally, on either side of the head (Rogers and Kaplan 2006). Consequently, the optic axes are directed outwards, instead of in parallel as with primates. The lateral placement of their eyes provides birds with a small binocular field of view and a large monocular field of view (Martin 2007, 2009). However, there is considerable species variation in the size of the monocular visual field, the extent of binocular field overlap (where the visual fields of both eyes converge), the blind areas, and the overall visual coverage (Jones et al. 2007; Martin 2014).

Amongst passerines, corvids have the broadest field of view, with the New Caledonian crow (*Corvus moneduloides*) possessing the largest (Troscianko et al. 2012). Specifically, New Caledonian crows are renowned for being one of a few bird species that forages by extracting grubs from tree cavities creating stick tools, which are held by their bill tip and pressed against their cheek for ease of manoeuvrability (Hunt 1996). Their visual field allows the crow to see their tools and the space that the tool is operating in, thus enabling them to operate the tool effortlessly, unrestricted by any visual blind spots (Kenward et al. 2011; Rutz et al. 2018; Troscianko et al. 2012). The lateral position of a bird’s eyes means that each individual eye has access to a completely different visual scene. Monocular vision allows birds to observe things with one of their laterally positioned eyes. Birds tend to observe events with one eye (either the left or the right) depending on the nature of the event (Koboroff et al. 2008; Rogers and Kaplan 2006). They are highly specialised in processing information differently based on which eye perceives it. Each eye transmits information contralaterally to the brain, with each hemisphere dedicated to distinct functions. The right hemisphere focuses on novelty and assumes control during distressing events, while the left hemisphere processes learned categories in more relaxed environments (Rogers 2008).

When experiencing an event, a bird must decide which zone of its visual system to focus on. This aspect of a bird’s perceptual system can lead to attentional blind spots and errors. For instance, blue jays (*Cyanocitta cristata*) exhibit reduced attentional performance when faced with complex foraging tasks (Dukas and Kamil 2000, 2001). Specifically, their ability to detect peripheral targets significantly diminishes during cryptic foraging tasks, likely due to the increased demand for focused spatial attention required to complete these challenging tasks.

Whilst corvids, like most birds, rely on vision as their main sensory modality (Birch et al. 2020; Jones et al. 2007), this family of birds also possess acute auditive faculties, which they appear to be able to use for a wide range of behaviors. Songbirds, such as the corvid family, are oft characterized by their complex and elaborate signaling system, which has been observed to be transmitted through social learning (Beecher and Brenowitz 2005; West and King 1996). Indeed, for most songbirds, their songs play an important role in many of their day to day lives such as reproduction, predator detection, territorial defense, courtship and social cohesion (Byers and Kroodsma 2009; Janik and Slater 2000), thus making the ability to accurately capture and discriminate these calls imperative for the survival and reproduction of the receiver. Consequently, the corvid family, as with most songbirds, possesses a sophisticated auditive ability. For instance, carrion crows have been shown to be able to discriminate between reliable and unreliable conspecifics based on their alarm calls alone (Wascher et al. 2015). Furthermore, crows have also been shown to be able to form and remember auditory categories (Wagener and Nieder 2020). After training, carrion crows showed proficiency in a delayed match to category exercise, which entailed the crows matching a particular remembered sound to two different categories with reference to the sound’s frequency (either upshifting or downshifting). Thus, evidencing their ability to store and retrieve such auditive categories in their working memory (Wagener and Nieder 2020). Alongside evidence from carrion crows, research by Shaw and Clayton (2014) shows that Eurasian jays can use and remember acoustic information when locating caches to pilfer from. Specifically, jays retrieved more caches after having had the chance to listen to their conspecific cache in both noisy gravel or quiet sand than when not being allowed to listen to the caching conspecific. Moreover, the jays also significantly searched more for caches to pilfer in the noisy substrate they heard a cache being made in (i.e., gravel), rather than in the quiet one they did not (i.e., sand) (Shaw and Clayton 2014).

Lastly, although olfaction is heavily used by mammals and reptiles (Meisami and Bhatnagar (1998); Moulton (1967); Taniguchi et al. (2011)), the olfactory ability of birds, particularly passerines such as the members of the corvid family, has traditionally been disregarded and thought to be sub-par by many (Clark et al. 1993). However, birds possess a fully working olfactory system, which also includes specific brain structures that parallel those of other vertebrates with good olfactory capability (Clark et al. 2015) and have distinctive plumage odours, which has been evidenced to inform others on the individual’s identity (Bonadonna and Mardon 2013; Moreno-Rueda 2017). The early assumption that members of the corvid family did not possess acute olfaction abilities is understandable, after all corvids show a small development of their olfactory bulbs in contrast to other species of birds (Bang 1971; Cobb 1968). However, evidence suggests that, whilst perhaps not as developed as neither their visual nor their auditive modalities, members of the corvid family can use olfaction during foraging and cache recovery. Specifically, in a series of field experiments, black billed magpies (*Pica hudsonia*) significantly uncovered more caches of food that was doused with highly scented cod liver oil, than the food that was not scented (Buitron and Nuechterlein 1985), and more seeds with natural scent or doused in gasoline than unscented plastic seeds (Molina-Morales et al. 2020). Similarly, ravens, have been evidenced to be able to locate fish only by using olfactory cues (Harriman and Berger 1986). Alongside cache recovery, Wascher et al. (2015) investigated whether carrion crows responded to the distinct odor of their conspecifics, and whether they preferred to orient themselves towards the scent of known, rather than unknown individuals. The tested sample of crows showed a significant avoidance to the conspecific smell. This result might be attributed to the extraction process of such smell, which caused stress to the bird, thus possibly resulting in the odour presented to the crows emanating cues of stress and thus causing the avoidance behaviour observed. Alongside this, the crows tested also displayed a significant tendency to prefer the odour of familiar individuals in contrast to unfamiliar ones. Overall, the evidence presented here suggests that some species of the corvid family exhibit sophisticated olfactory systems, which they can use to locate, and discriminate potential food items and individuals. However, more evidence is needed to further explore the olfactory abilities of this family of birds.

### Evaluative richness

Evaluative richness (or as Birch et al. also call it: ‘e-richness’) refers to the richness of the experience by an animal of their positively and negatively valenced states; how the animal represents their own evaluation of these states as positive or negative for itself. This dimension encompasses a range of affective experiences with positive and negative valence, such as the commonly discussed pleasure and pain, but also hunger, thirst, boredom, comfort, curiosity, and social bonds. Any animal capable of valanced experience will have at least minimal e-richness, however the shape and scope of this capacity will vary for different species.

In investigating the e-richness of an animal, a useful range of lines of evidence exist. One potentially promising line could be a direct assessment of the evaluation system and its mechanistic complexity. However, this is something we are still figuring out even in the human case and is likely to be of limited use for the study of nonhuman animals at this stage. Instead, we can look for the presence and complexity of different behavioural and cognitive abilities linked to evaluation. Here, we will assess the extent of this evidence for corvids, to build up a profile of their e-richness, from what we currently know about the taxon.

The first line of evidence—and perhaps the strongest—is the ability to make motivational trade-offs. That is, to assess and evaluate competing motivations and decide on a course of action that best satisfies current needs. The ability to do this flexibly as the strengths of different motivations change, and to assess a wide range of competing affects, would both be evidence of higher e-richness. Unfortunately, there is very limited work investigating this ability in corvids, and as such few conclusions can be drawn. One study on wild birds (ravens, hooded crows, and magpies) has shown that members of the less dominant species are more likely to leave cover to approach an unoccupied carcass—theorised as making a trade-off between hunger and risk of predation, where less dominant species are typically more hungry and thus more willing to take on the risk (Halley 2001). However, as this is closer to a wild observational study, rather than a controlled experiment with varying conditions, the exact mechanisms and explanation are unclear and this would serve as, at best, weak evidence of the ability to make motivational trade-offs.

Another line of evidence for e-richness in a species is the capacity for cognitive biases modulated by mood. These biases have been demonstrated in humans, and appear to result from an overall evaluation of the current or recent conditions an individual has experienced. Cognitive judgement biases, the most commonly tested type of bias, result in an optimistic or pessimistic judgement of an ambiguous stimulus, depending on the mood of the animal (typically inferred through an intervention intended to manipulate emotion, such as provision of positive or negative living conditions) (Lagisz et al. 2020; Mendl et al. 2009).

There have been a few studies using cognitive judgement bias to test the emotional states of corvids. New Caledonian crows have been shown to display optimistic bias after use of tools, suggesting that performance of such behaviour is positively valenced for them (McCoy et al. 2019). Ravens display pessimistic biases after observing a social companion in a negative mood state, suggesting their mood can be affected by their perception of the mood of others (Adriaense et al. 2019) (more on this shortly). These tests suggest that corvids have the evaluative capacities that underlie the development of cognitive biases. However, some caution is warranted here. The method of cognitive judgement bias testing has been used as a welfare indicator, looking to assess the welfare impact of different conditions. The underlying psychological mechanisms and features of the bias are not themselves investigated. More direct tests examining the features and functioning of the cognitive biases, including investigation of other types of cognitive bias such as attention bias (Crump et al. 2018), would give a richer picture of the animals’ evaluative capacities.

Evaluative richness can also be assessed through examining complex forms of learning, as this requires association of positive or negative value—reward or punishment—with different conditions or behaviours. Although simple associative learning of this type is too widespread to tell us much about an individual’s mind, particularly strong evidence comes from reversal learning, in which an individual can learn to switch the value of previously learned stimuli—a formerly rewarding stimulus becoming a punisher. This is suggestive of a stronger and richer evaluative capacity. Several species of corvid—crows, pinyon jays, Clark’s nutcrackers, Florida scrub jays, and western scrub jays—have been successful in demonstrating reversal learning, including serial reversal learning, where the stimulus-reward pairings are reversed multiple times (Bebus et al. 2016; Bond et al. 2007; Teschke et al. 2013; Wascher et al. 2021). It is not clear, however, whether corvids’ performance is necessarily exceptional amongst the birds. Some studies found that domestic chickens and Galapagos woodpecker finches performing just as well as crows when tested alongside them (Teschke et al. 2013; Wascher et al. 2021); whereas other researchers found that corvids showed significantly better performance than chickens, quails, and even one species of parrot (Gossette et al. 1966).

We can also look for evidence of the more cognitive, rather than affective, side of the evaluation—whether and to what degree animals are capable of assessing the value of markers of reward, such as tokens, or the comparative value of different food items. Corvids have succeeded in token-exchange experiments, demonstrating their ability to perform such evaluations and distinguish between different tokens (Wascher et al. 2019; Wascher and Bugnyar 2013), as well as several food-exchange paradigms (Dufour et al. 2012, 2019; Hilleman et al. 2014; Wascher et al. 2012). They are capable of delaying a reward in favour of a greater future payoff up to several minutes in the future, weighing eventual reward value against time required to wait (Dufour et al. 2012), though only when it is an increase in reward quality, not quantity (Hilleman et al. 2014; Wascher et al. 2012). However, they appear incapable of calculating odds of risky choices with variable reward payoffs, instead sticking to single exchange strategies throughout (Dufour et al. 2019). Interestingly, they have also demonstrated inequity aversion, refusing to work more often when a conspecific was observed to be rewarded for ‘free’, suggesting quite a sophisticated capacity for evaluating the efforts and payoffs for themselves and others (Wascher and Bugnyar 2013).

Regarding the qualitative nature of corvid evaluation, we can look to see which affects are present, and which more strongly dominate their experience (i.e. build up their affect profile, Browning 2022). Unfortunately, current research on specific affects in corvids is severely lacking, but there are some interesting possibilities for understanding corvid affects, many of which centre around social interactions. Bird brains contain neurochemicals (dopamine, opioids) associated with the experience of pleasure in mammals, and their presence in analogous brain regions suggests they play a similar role (Riters 2011; Emery and Clayton 2015). It is possible that corvids can experience fun, demonstrated in part by play behaviour—seemingly rare among birds, but present in corvids (Emery and Clayton 2015), and young ravens show ‘play contagion’, increasing their level of play when observing another bird playing (Osvath and Sima 2014). More generally, ravens seem capable of ‘emotional contagion’—where an individual’s mood is influenced by that of its peers. Ravens that observed a conspecific in a negative mood appeared to take on a negative mood themselves (Adriaense et al. 2019). This is suggestive of the presence of prosocial or ‘empathetic’ affects, involving some positive or negative valence associated with the pleasures or sufferings of others. The observed changes could also simply represent the animal’s sensitivity to the possibility of reward or punishment arising from the observation of the wellbeing of another bird (Vonk 2019)—though it is worth noting that this in itself is a somewhat sophisticated evaluative ability.

Corvids may show ‘consolation’ behaviour, with ravens demonstrating affiliative behaviour to the victim of a conflict they were not themselves involved in, hypothesised to reduce the distress of the victim (Fraser and Bugnyar 2010b). They show a range of affiliative behaviours, primarily within long-term monogamous pair-bonds, such as mutual preening, food sharing, bill-twining, contact sitting, and maintaining close proximity when feeding and playing, suggestive of prosocial or bonding affects (Clayton and Emery 2007a). Corvids show unique responses to dead conspecifics, including vocalising, gathering around the dead birds, making occasional tactile contact, and showing wariness in areas associated with death; though these appear more like responses to danger than of anything like grief for the dead birds—the response to the death of familiar individuals is unknown (Swift and Marzluff 2015, 2018). All of these observations are suggestive of the presence of social affects, but more work is needed to determine the nature and strength of these experiences.

Thinking about e-richness also involves considering how an animal evaluates the world around it—their ‘style’ of evaluation. For corvids, a large part of their evaluative style is shaped by neophobia—wariness around and avoidance of novel stimuli, to a degree greater than that of other bird species (Brown and Jones 2016; Greggor et al. 2016; Miller et al. 2022). It is a surprising trait, given their propensity for innovation and behavioural flexibility and the generalist lifestyle of many corvid species. It may be related to greater learning flexibility, such as in performance on reversal learning tasks (Bebus et al. 2016) and correlates with socioecological factors, including presence in urban environments, sociality, flock size, and caching behaviour (Miller et al. 2022).

Taken together, there is at least preliminary evidence that corvids have a high degree of e-richness. However, without a more explicit scale of how to assign high or low scores, it is hard to determine exactly, or how they compare to other species. We have a better sense of the features of evaluation, what evaluative experience is like for the animals: a more qualitative assessment. Future work identifying the components of evaluative richness and how well corvids fulfil them could help make clear how to comparatively score e-richness for these and other species.

### Synchronic unity

Birch et al. discuss unity as the integration of experience *at a point in time* into a single unified sphere or a unique point of view. Consciousness in humans appears to be highly unified and many have argued that unity is a necessary component of consciousness, with proponents of the Integrated Information Theory of consciousness (IIT), even making it *the* defining feature of consciousness (see Tononi 2004, 2005, 2008, 2010, 2012a, b, 2015; Balduzzi and Tononi 2008, 2009; Tononi and Koch 2008; Tononi et al. 2016; Koch and Tononi 2011).Whether consciousness must be unified in non-human animals, however, is not quite as clear. After all, the possibility of disunified streams of experience is already being taken seriously in human patients with so-called ‘split brains’ (Volz and Gazzaniga 2017; Pinto et al. 2017; de Haan et al. 2020). As Birch et al. 2020 note, humans in which the *corpus callosum* is “wholly or partially severed” show disunified behaviour suggestive of two distinct streams of consciousness when information is being presented in only one of the visual fields (p. 793). Furthermore, Birch et al. (2020) suggest that birds could serve a natural experiment of split-brain patients since they have no brain structure functionally analogous to the *corpus callosum*. However, here we think that this analogy has been overstated (see also Veit 2021, 2023 for a longer discussion of the relationship of consciousness and synchronic unity, from where parts of the review in this section are recapitulated from in improved form, and we move further here to consider how synchronic unity is related to the other dimensions). Importantly, the lower regions of bird brains are connected and make up a larger part of their brain than the cortex does in humans (Vallortigara 2000). Rather than thinking of their experience as one or two streams of experience, corvids offer an interesting case of partial unity. Although one hemisphere typically appears to dominate for particular tasks, there still appears to be plenty of information exchange between the hemispheres. For instance, corvids seem to have a spatial memory tasks preference of the right eye object-specific cues whereas the left eye seems to focus on spatial cues (Clayton and Krebs 1994) and New Caledonian crows have even been shown to have foot preferences for tool use/manufacture (Weir et al. 2004), which suggests possible adaptive benefits of disunity.

The evolution of both birds and mammals is a striking example of increased lateral brain connectivity. For this reason, we should be cautious about portraying birds as a paradigm of disunity. Their descent from theropods—a clade of dinosaurs that includes all predatory species—likely reflects an increase in the unity of consciousness. This makes non-avian reptiles a more suitable case for exploring the possibility of disunified experience.

Birch et al. (2020) consider electroencephalograph studies of sleep as further evidence challenging the necessity of unity because birds have also been shown to engage in unihemispheric sleep. If two streams of consciousness can coexist, one could imagine a scenario in which an animal experiences dreams in one part of its brain while the other remains awake and actively assessing its surroundings. While further research is needed to explore such possibilities, the existence of functional specialization within brain hemispheres suggests that consciousness does not necessarily have to be unified. Intraocular and interocular transfer studies in pigeons (*Columbia livia*) by Ortega et al. (2008), have inspired Birch et al. (2020) have argued that they may have limited visual integration between the yellow field of the retina for the lateral monocular field and the red field responspible for the lower frontal region (pp. 793–794). Curiously, almost all pigeons failed to have interocular transfer between the yellow fields of each eye, whereas there is such transfer between the red fields (Ortega et al. 2008). This potentially undermines the need for conscious vision to be presented in a unified field (see also Hill 2018). Could such partial unity offer an adaptive advantage? For birds with lateralized eyes and limited visual overlap, this possibility is far from unreasonable. Each eye could be engaged in a distinct task, such as predator detection versus foraging, potentially providing a benefit over the singular focus of attention that unified consciousness appears to require in humans. Future research should investigate this capacity in corvids to explore potential variation across the avian class. A very recent article has shown lateralization of the first high-level excecutive control mechanisms in crows for volitional visual attention (Hahner and Nieder 2025), which is better evidence to examine the synchronic experience of species since high-level processes are more likely to involve conscious experience than lower-level ones. As we learn more about how often corvids are engaged in mental division of labour in the wild, this experimental research will allow us to make more confident predictions about whether the subjective experience of corvids is unified or not.

The study of unity has many links to the other dimensions. In thinking about the evolution of subjective points of view, we are interested in the integration of sensory experiences into a single field. Furthermore, there are direct links to evaluative experience. Since rich forms of associative learning are suggestive that distinct stimuli are experienced together to allow for learning, complex evaluation is highly suggestive of synchronic unity. This is where the idea of a ‘common currency’ is useful since it suggests that distinct sensory experiences are associated with a single dimension of valence (positive and negative) that can be traded off against each other in something like instantaneous affective decision-making (Veit 2022). Selfhood is also straightforwardly related to unity since a sense of self relies on the integration of sensory stimuli. Lastly, temporality may also help us to make a better sense of unity since it is integration across time (hence diachronic unity). Studying these dimensions may help us to make sense of the functional role diachronic unity has for conscious animals. Finally, although the evidence on this dimension is hardly conclusive, corvids could constitute a good case for something like partial integration. Further studies of division of labor in the corvid brain will be necessary to investigate this further.

### Diachronic unity

As Birch et al. (2020) discuss, typical human consciousness is extremely integrated across time. We experience a continuous stream of consciousness, flowing seamlessly from moment to moment (Dainton 2010; James et al. 1890). As mentioned, corvids must interact with the world whilst flying at high speeds; weaving through obstacles to hunt for fast moving or elusive prey, or to evade equally agile predators. Subtle changes in the position and movement of these objects and agents must be accurately perceived, and inferences need to be made about their trajectories, as well as the birds’ own. As such, experiencing events around them as a temporally integrated stream would, ostensibly, be highly adaptive or even essential for survival.

Birch et al. (2020) examine the use of the colour-phi illusion as a tool for investigating mechanisms that alter sensory input of static stimuli into a continuous, coherent stream in humans. In the illusion, two different coloured spatially separated dots are perceived as a single moving dot (that changes colour) when the dots are flashed in sequence (Kolers and von Grünau 1976). As such, the brain does not just mistake the two static stimuli as one moving stimulus, but also forms an explanation of how the stimulus changes. Pigeons (*Columba livia domestica*) have been behaviourally demonstrated to be able to detect apparent movement (Siegel 1970), the perceived movement of a stimulus that is not in actual physical motion, and are unable to differentiate between apparent movement and real movement (Siegel 1971). Analogous to the colour-phi illusion, pigeons were shown to perceive a sequence of projected static images of black or white strips as a single strip moving and changing colour. Furthermore, as reviewed above, birds distinguish between light that they perceive as blinking vs continuous, indicating that their subjective visual experience forms a continuous stream above a certain threshold of flicker-fusion frequency. Whilst these processes have not yet been shown in corvids, as discussed, avian visual systems seem to be deep set within the taxa, making the inference that corvids share this perceptual phenomenology likely.

As well as short-term unity, Birch et al. (2020) discuss temporality across the long-term, referring mainly to mental time travel (MTT). Human experience is not limited to the here and now, or exclusively dictated by external perceptual events. We often drift into stimulus-free, offline thought, directed towards our past or our future. MTT is this ability to travel through our subjective time, reconstructing past personal experiences as well as imagining possible futures. MTT is essential for a standard, temporally integrated human experience of living, as demonstrated by patents suffering from hippocampal amnesia such as Clive Wearing (Suddendorf et al. 2009).

Wearing experiences life as blinking from moment to moment, perpetually declaring ‘I am now awake’ (Wearing 2005). Without reference to the past (near or distant), Wearing is bound to current perceptual stimuli, before this too is lost to the void. These observations, amongst others, lead to the delineation of episodic memory (EM) (memory involving the conscious recollection of personally experienced events) within the declarative memory system (Tulving 1983). As well as losing his ability to engage in EM, Wearing, like other amnesic patients with analogous hippocampal damage, is unable to think about the future (Corballis 2014; Hassabis et al. 2007; Klein et al. 2002; Rosenbaum et al. 2005; Suddendorf et al. 2009; Tulving 1985).

MTT is often thought to be a uniquely human ability (Suddendorf and Corballis 1997; Suddendorf and Corballis 2010; Tulving and Markowitsch 1998; for a recent review see Davies and Clayton 2024). Whilst many non-human species show sophisticated memory abilities and anticipatory behaviours, some researchers have argued that they cannot consciously recall specific past experiences and imagine future scenarios as humans can (Suddendorf and Corballis 1997; Tulving and Markowitsch 1998). However, evidence for MTT and its characteristic phenomenology in humans is centred around language-based reports. Using Tulving’s (1972) original definition of EM, stating that it “receives and stores information about temporally dated episodes or events and temporal-spatial relations among these events” (p. 385), Clayton et al. (2001a, b) argue that simultaneous retrieval and integration of information about the ‘what’ and ‘when’ of unique experiences (‘temporally dated experiences’), and ‘where’ they occurred (‘temporal-spatial relations’) represents the behavioural characteristics of human EM. This ‘what-where-when’ paradigm allows EM to be investigated in non-linguistic animals, but in the absence of evidence for the associated conscious experience, the ability is termed ‘episodic-like memory’ (ELM) (Clayton and Dickinson 1998).

In a seminal study, corvids (western scrub-jays, *Aphelocoma coerulescens*) have been demonstrated to fulfil the criteria for ELM (Clayton and Dickinson 1998). As jays store non-perishable foods (e.g., nuts) and perishable foods (e.g., insects) for future consumption, it is argued that ELM has evolved in these birds to facilitate remembering the contents, location, and timing of their caches, and therefore recover foods before they become inedible (Grodzinski and Clayton 2010). By exploiting this natural phenomenon, this study demonstrates that jays are recalling the ‘what’ (food type), ‘where’ (cache location), and ‘when’ (time of caching relative to retrieval), when recovering trial-unique caches after different intervals. Eurasian magpies (*Pica pica*), another corvid, have also exhibited the use of what-where-when memory (Zinkivskay et al. 2009).

Whilst intended to bypass the question of conscious experience, research with humans shows that we do employ phenomenologically complete EM during what-where-when tasks that are designed to replicate the conditions of animal studies as closely as possible (Holland and Smulders 2011). Furthermore, additional experiments investigating the what-where-when memory system in scrub-jays may suggest a more conscious experience depending on integrated and manipulatable representations of what-where-when information (Clayton et al. 2001). Clayton et al. (2003a, b) argue that whilst the individual what/where/when components could be learned for an event, the representation may not be fully integrated in a manner that would allow for the retrieval of all the elements from the presentation of a single element. If not fully bound, two events that share the ‘what’ and ‘where’, but differ in their ‘when’, could not be discriminated between. Therefore, the what-where-when information must be bound together in an integrated structure, consequently yielding a distinctive and unique memory. Furthermore, as human episodic memories can freely interact with semantic information to generate behaviour in a flexible manner (Tulving and Markowitsch 1998), an animal must be able to use the what-where-when information regarding an event in an adaptable manner that is not predetermined during encoding, and thus can interact with novel semantic information. Scrub-jays were demonstrated to form fully integrated what-where-when memories (Clayton et al. 2001a, b) and were able to flexibly use novel information, given after the original caching event, to dictate their recovery behaviour (Clayton et al. 2003a, b).

That said, the repeated training in these experiments, necessary for the subjects to learn the temporal ‘rules’ of the experimental sequence (e.g., specific degradation rates), has led to some authors arguing that non-episodic solutions are available to solve these tasks (e.g., Crystal 2021; Davies et al. 2022; Zentall et al. 2001). With repeated exposure, the animals in these studies have the opportunity to learn to explicitly encode and retain certain information within an event for use in an anticipated memory test, and as such, may preselect a future action based on a non-episodic memory trace (Crystal 2021; Roberts et al. 2008; Zhou and Crystal 2009). However, corvids (Eurasian jays, *Garrulus glandarius*) have also recently been demonstrated to show evidence for episodic-like memory through an alternative paradigm (Davies et al. 2024a, b) that forces subjects to recall back to an encoding event upon the presentation of an unexpected memory assessment (a central hypothesis surrounding episodic(-like) memory (Crystal 2013, 2021)).

In these tests, known under the incidental encoding and unexpected question paradigm (Zentall et al. 2001), a subject is exposed to various details within an encoding event, some of which are never targeted as relevant (i.e., associated with rewards) and thus represent ‘incidental’ information. If the animal is subsequently able to use this information to ‘solve’ a memory test, which they are not trained to expect, this suggests that this incidental information, despite not being marked as important enough to explicitly encode into memory, is automatically encoded within the memory (a characteristic of human episodic memory (Morris and Frey 1997; Zentall et al. 2001)) and is retrieved upon presentation of the memory test. Importantly, to be able to access this incidentally encoded information for use in said memory test, the subject must recall a holistic representation of the event, containing a plethora of details surrounding the episode including the narrative sequence, and subsequently replay, target and manipulate this information within the event representation (Crystal 2021; Davies and Clayton 2024; Davies et al. 2024a, b; Panoz-Brown et al. 2018; Sheridan et al. 2024). Although this may occur in the absence of conscious experience, recent evidence reveals that human subjects have conscious access to incidentally encoded information within their memories and can target these details in order to solve a memory assessment (Kaunitz et al. 2016; Matthews et al. 2018, 2019; Qianchen et al. 2022), therefore raising the possibility that corvids (as well as other songbirds; Davies et al. 2024a, b) may also have access to incidental information within remembered events (Davies and Clayton 2024).

Although this possibility remains, neurophysiological divergence between bird and mammal brains may suggest that their subjective experience of episodic-like memory may differ, potentially being diminished in avian taxa (Davies & Clayton 2024). Whilst research suggests that in mammalian brains, the direct connections and synchronous activity between the hippocampus and the neocortex allow for close interactions and information pathways between these structures (Manns & Eichenbaum 2007; Simons & Spiers 2003), the avian hippocampus, although vital in the processing and storage of spatial information contributing to episodic-like memories (Bingman et al. 2005; Colombo & Broadbent 2000; Sherry 2006), does not appear to receive a comparable input from its analogous high-order cortical areas (Rattenborg & Martinez-Gonzalez 2011). Consequently, components of daily experiences (processed within these regions) may not contribute to episodic-like memory formation and retrieval in birds in the same way as in mammals (i.e., humans), and accordingly, their experience of episodic-like may be phenomenologically limited in comparison (Davies & Clayton 2024).

Behavioural paradigms have also been utilised to assess the future planning abilities of non-human animals, including corvids. As discussed above, carrion crows (*Corvus corone*) and ravens (*Corvus corax*) appear to assess if a future exchange is worth waiting for, by abstaining from collecting a reward for several minutes in order to obtain a better reward (Dufour et al. 2012; Hilleman et al. 2014). Scrub jays seem to plan for different future scenarios by caching in locations where they expected to not receive food the next morning (Raby et al. 2007), although these results did not replicate with another corvid species, the Canada jay (*Perisoreus canadensis*) (Martin et al. 2021). Evidence from experiments exploiting specific satiety, the decline in preference for a particular food when it is eaten relative to a food that has not been eaten, suggest that Eurasian jays can plan for future needs that contradict their current motivational states (Cheke and Clayton 2012).

To pass the ‘spoon test’ Tulving (2005), an individual, having previously seen a specific task apparatus, must collect an appropriate tool from a separate location and retain it, knowing it may have the opportunity to use it in the future. Therefore, the test requires subjects to retain representations of different possible future scenarios, based off previously acquired information, and perform specific actions at present to plan for these scenarios (in the absence of situational cues relating to the original episode). A preregistered experiment, conducted by researchers on opposing sides of the ‘human uniqueness of MTT debate’, Boeckle et al. (2020) demonstrate that New Caledonian crows (*Corvus moneduloides*) pass the spoon test, and thus can plan for specific future events. First, the crows were trained to obtain rewards from three different apparatuses using three different tools (sticks, hooks, and stones) that could only be used with their respective apparatus. Next, they were trained on the experimental sequence: Stage 1: birds observed a baited ‘stick apparatus’ in a compartment; Stage 2: after 5 min, in a different compartment, they were allowed to choose between 5 objects including the three tools; Stage 3: 10 min later, the crows were returned to the original compartment with their chosen object and were allowed to collect the reward (if they chose the stick). During training, the only sequence the birds experienced was the condition with the stick as the correct tool. However, during Stage 1 of the test phase, the crows were presented with one of the other two apparatuses. Consequently, until the test phase, the birds never experienced the experimental sequence in which the hook or stone tools were the correct choices. To solve the test trials, the crows had to utilise their memory of which apparatus they observed during Stage 1, whilst ignoring the object that was the most associated with food rewards (the stick).

It must be said, however that all the aforementioned studies investigating MTT in corvids have been conducted using paradigms which exploit highly specialised natural behaviours. Therefore, the extent to which these abilities are generalised across contexts, and therefore more like conscious human MTT, is unknown (Suddendorf and Corballis 2010; for a recent review see Davies and Clayton 2024). As such, future research should explore MTT in corvids outside of an evolutionary context.

### Self-consciousness

Selfhood, sometimes referred to as ‘self-awareness’ or ‘self-recognition, and Theory of Mind (ToM) are often grouped together because they may both play a role in self-other awareness. As stated in Birch et al. (2020), “like all the other dimensions, this is a capacity that admits of gradations” (p. 797). A tentative definition of selfhood is the ability to recognise oneself and one’s body as distinct from the rest of their environment. By contrast, Theory of Mind,is the ability to attribute mental states to oneself and to others. The term was first coined by Premack and Woodruff in (1978) when studying the cognitive capacities of chimpanzees. But is there any evidence for these two abilities (selfhood and ToM) in animals?

One commonly used procedure to study self-recognition in animals is the mirror self-recognition (MSR) test developed by Gallup (1970). Prior et al. (2008) found some evidence of MSR in magpies by placing a brightly coloured mark on the front of their bodies. Since then, other researchers have attempted to test the self-recognition capacities of other corvids species (e.g. Baciadonna et al. 2020; Soler et al. 2014). However, it is important to note the criticisms surrounding MSR studies (e.g. Hillemacher et al. 2023). At a base level, there stands the question of whether the individual views the reflection as self or other. This is why some researchers propose selfhood and theory of mind are linked, as there is so much more to this specific dimension of consciousness than awareness of one’s own body; it is also important to take other’s bodies into account. Therefore, it is possible there are simpler explanation for the results seen in many MSR studies, such as an understanding of mirror guided body movement (see Clary and Kelly 2016). This doesn’t mean that MSR studies are necessarily flawed, but rather that it is unclear the extent to which tests involving mirrors actually measure selfhood or awareness and, therefore, it is important to be cautious when interpreting the results. The same can be said of ToM studies.

In Premack and Woodruff’s (1978) seminal study in which they first coin the phrase’theory of mind’, the primary criticism was whether Sarah the chimpanzee really understood the beliefs and desires of the experimenters or whether she had simply learned to associate certain action with certain outcomes. This led to much research, initially focused on chimpanzees, testing whether the animals really understood the perceptions, beliefs, and knowledge of others be they human experimenters or conspecifics. The evidence was mixed and interpretations controversial. For example, Povinelli’s research suggested that chimpanzees do not have ToM even if they can learn associately over many trials to distinguish behaviourally between one that is knowledgeable (the knower) and one that is not (the guesser) (e.g. Povinelli, Nelson, and Boysen Povinelli et al.; Povinelli 1994; Povinelli et al. 1994). This work was criticised in terms of lack of ethological validity. Chimpanzees in the wild don’t normally interact with humans, especially those wearing a bucket over their heads. Additionally, in their day to day lives with conspecifics, food interactions are of a competitive nature, not a cooperative one (Call 2007; Emery and Clayton 2009). In response to these criticisms, Hare et al. (2001), developed the competitive food paradigm in dyadic interactions with a dominant and subordinate chimpanzee to test whether the subordinate chimpanzee would change its decision of whether or not to collect hidden food depending on what the dominant chimpanzee knew. By and large, the subordinate chimpanzees were able to pass these tasks; however, at issue is whether they were ‘reading the mind’ of the dominant chimp (knowledge attribution, which is a type of ToM) or whether they were merely responding associatively to their behaviour. Almost half a decade later, researchers continue to study ToM throughout the animal kingdom in species such as primates, canines, and, of course, corvids (Drayton and Santos 2013; Maginnity and Grace 2014; Krupenye and Call 2019).

Like many primates, some corvids, such as jackdaws, have been known to respond to gestural and gaze cues of human conspecifics in an object-choice task, a phenomenon associated with ToM (Emery and Clayton 2009; von Bayern and Emery 2009). ToM has also been studied using desire-state attribution in Eurasian jays; male jays fed their female partners the food their mates wanted to eat, but only when they had previously observed what their partner had eaten. This suggests that the male jays assessed what their female partners wanted to eat by observing what they actually are as opposed to simply responding to behavioural cues such as their mate’s attention to the foods the male might provide them with (Ostojíc et al. 2013). Observation of caching and pilfering behaviour (the storage of food by and later retrieval of food) is another common method of studying ToM in corvids (Reznikova 2007; Vander Wall 2003). Corvids have developed many clever ways to conceal their caches from pilferers. Why? They’re usually the ones pilfering from other birds (Dally et al. 2006, 2010; Clary and Kelly 2011, 2013; Vernouillet et al. 2021, 2023).

Californian scrub-jays with pilfering experience will re-cache food if they know they have been observed by a potential pilferer and only birds with prior experience of pilfering will re-cache their food themselves; however, naive birds with no pilfering experience did not do so, suggesting these cache protection tactics rely specifically on the experience of being a thief (Emery and Clayton 2001). In other words, it takes a thief to know a thief. When caching, corvids are not only thinking about how best to protect food for themselves and their partners, but they are also using their knowledge as pilferers; they know some of the best cache protection strategies because they have stolen from some of these cleverly hidden caches themselves.

High sociality is often associated with higher cognition rates and social living is one of the explanations for the evolution of social cognition. (Humphrey 1976; Jolly 1966; Krupenye and Call 2019). Therefore, the presence of high cognitive functions such as ToM in a highly social species such as corvids should come as no surprise. Despite the negative connotations of the phrase’bird brain’, corvids have large brains in relation to their body size, and their brain-to-body ratio–or encephalisation ratio–is the same as chimpanzees. The two largest portions of the avian brain, the nidopallium and mesopallium, have often been compared to the mammalian prefrontal cortex, the part of the brain used for complex decision making (Güntürkün 2005; Clayton and Emery 2015). Whereas components such as a large brain-to-body ratio and sharp decision-making skills are perhaps responsible for the evolution of high intelligence in corvids and many of the mechanisms of ToM itself, there is a possibility the selective pressures of social living drove this evolution as well. Corvids usually mate for life and most species are social; however, the degree of this sociality varies. Some species, like American crows and Florida scrub-jays demonstrate communal-cooperative breeding, meaning the offspring are raised by more than two individuals; while both the male and female provide care for their young, they are also assisted by non-breeding relatives, called ‘helpers’ (Bresgunova 2016; Clayton and Emery 2007b; Cockburn 2006). Although rooks do not participate in communal cooperative breeding, they forage, roost, and nest together as flocks (Seed et al. 2008). Ravens, by contrast, are much more solitary and territorial but travel in pairs with their mates (Fraser and Bugnyar 2010a).

Even solitary species such as ravens still require complex cognitive functioning. Whereas corvids living in social groups must learn to interact and co-exist with other conspecifics to survive, less social species must learn how to protect themselves, as well as their mates, territories, and caches (particularly from conspecifics). The need to socially interact with others could have been the environmental pressure that facilitated the evolution of ToM (Jolly 1966; Humphrey 1976; Brüne and Brüne-Cohrs 2006). Regardless of whether the social interactions a particular corvid experiences with a conspecific are more amicable or antagonistic, these interactions require an understanding of not only oneself but also of others.

It is important to note that, despite the information presented above, there is no clear yes or no answer regarding whether corvids possess ToM. There are certainly sceptics (e.g. Penn and Povinelli 2007), many of whom believe that no study in particular has shown any unequivocal proof for ToM in animals because it is impossible to empirically distinguish between mind-reading and behaviour-reading explanations in moist if the studies that have been conducted. The one possible exception is in the case of scrub-jays recaching hidden food stores that observers have watched them cache, once those potential pilferers have left the scene since, at the time of recaching, the birds are not responding directly to the behaviour of the potential pilferers as those birds are no longer present (Emery and Clayton 2001; Dally et al. 2006). It is also worth noting that absence of evidence is not evidence of absence, and it is perhaps the converging evidence of all these studies together that provide more compelling evidence than any one study on its own. ToM research is constantly evolving, and it doesn’t seem to be slowing down. Further studies in the field, specifically those utilising Tinbergen’s four levels of analysis (causation, survival value, ontogeny, and evolution; Tinbergen 1963) hope to uncover more about the nature and origin of ToM (Krupenye and Call 2019;). By doing so, we can hope to learn more about this fascinating dimension of consciousness, its evolutionary origins, and how our animal relatives use it.

## Conclusion

Our goal in this paper was to make use of the framework first introduced by Birch et al. (2020), distinguishing five different dimensions of animal consciousness, to advance an empirically informed approach to the question of what it is like to be a corvid. It was not our intention to provide an argument for corvids being conscious at all, but rather to explore what the features and varieties of this experience may look like if they are. As we hope to have demonstrated, corvids have a variety of skills and cognitive capacities that are suggestive of some level of richness across all five dimensions of consciousness. It is difficult to assign precise values to these dimensions without further development of the framework to identify what such comparative scores would look like, but here we have provided a more qualitative survey of what the conscious experience of corvids is likely to be, across each of these dimensions. It is clear, however, that they particularly stand out—not only among other animals, but also in comparison to humans—in their temporal richness. For this reason, they can serve as a model taxon for the comparative study of animal consciousness on this if not the other dimensions.

Yet, it is also clear that there is a lack of research within some of the dimensions of corvid consciousness. For instance, we noted that there is very little research on olfaction in corvids, as well as on experience of selfhood. Perhaps most important, there is a lack of research on evaluative richness. Because this dimension is arguably the most important one for considerations of animal welfare, more research is needed—e.g., on motivational tradeoffs and the types of affects these birds experience—to better understand this dimension of corvid consciousness. Expanding research into the dimensions of corvid consciousness and increasing understanding of how they experience the world is important both for building knowledge and for social and ethical applications. Although, as we have noted, we did not intend to provide an argument for the consciousness of corvids, we believe that the evidence is sufficient to consider them to be plausible sentience candidates (Birch 2024). This means they should fall under the scope of an animal precautionary principle such as that advocated by Birch (2017, p. 4): “Where there are threats of serious, negative animal welfare outcomes, lack of full scientific certainty as to the sentience of the animals in question shall not be used as a reason for postponing cost-effective measures to prevent those outcomes” and their welfare interests should be protected. Research into the dimensions of consciousness for any animal also has important implications for their welfare (Dawkins 1980), as well as for policy or guidelines aimed at their protection. Understanding the consciousness profile of corvids helps us determine how best to care for or protect them and to identify what they want and need.

As mentioned, much of this will come from the evaluative dimension, as this is the seat of the valenced experiences most relevant to welfare (Duncan 2002; Browning 2020; Mellor et al. 2020). For instance, we know that they enjoy performing species-specific behaviours such as tool use (McCoy et al. 2019) and so these opportunities should form a part of good husbandry for positive welfare. They are highly social and possibly empathetic to conspecifics, and so it is important to house them in groups and prevent them, when possible, from witnessing the negative experiences of others. They are highly neophobic, so any new objects or other changes to the environment should be introduced slowly and carefully. For example, understanding the neophobia of ʻalalā, a Hawaiian crow, has implications for how to implement conservation breeding and release projects (Greggor et al. 2020). Building an understanding of the affect profile of the different species will help in understanding where the greatest risks or contributions to welfare might lie.

However, there are also potentially relevant welfare aspects found within the other dimensions. Understanding sensory richness will help to identify what parts of their sensory environment they are likely to attend to and to what degree, so that housing and husbandry can be modified accordingly. For instance, the high visual and auditory acuity of corvids may make them sensitive to unpleasant or threatening stimuli that humans would fail to notice; and their often-overlooked olfactory capacities could form the basis for the use of a range of olfactory enrichments. When thinking about synchronic unity, especially the obvious degree of lateralisation demonstrated in corvids (and other birds), this can affect not just the perceptual but also emotional processing of stimuli (Goursot et al. 2021), with potential welfare impacts. Understanding temporal unity will tell us about how important memory and planning are to the animal, and about the potential ongoing welfare impact of negative experiences. For corvids, their strong capacities for episodic-like memory and future planning should make us particularly wary of uses or processes that will inflict acute suffering. They are less likely than other groups of animals to simply forget, and may instead experience ongoing negative effects. Finally, the experience of selfhood is likely to relate to the importance an individual places on its own agency, with agency, choice, and control emerging as crucial pillars of animal welfare (Špinka 2019). Additionally, what appear to be theory of mind capacities in corvids, particularly relating to their caching behaviour, should be taken into account when planning social housing and provision of visual barriers to retreat behind. Being unable to engage in caching activities without being observed could cause stress and suffering.

Finally, we hope that our use of the five-dimensional framework to approach corvid consciousness in a comparative manner will encourage research to address the diversity of minds in nature, rather than just asking whether a particular species is conscious. There are many taxa, most of which have received very little attention, but we hope that extending the kind of work we have reviewed here to other taxa will lead to a better understanding of consciousness in all its forms. Although this research can be speculative in its formation of new hypotheses, this places it in no worse a position than other fields with similarly difficult to target phenomena—such as paleobiology, or the social sciences—where alternative hypotheses often can’t be firmly ruled out by the available evidence. Just as this does not make those fields idle speculations, we hope to have shown here that the same can be true for an expansive study of animal consciousness; within this science we can see that progress is being made, tests are being developed, and the animal mind is being demystified. This research is difficult and faced with methodological and conceptual problems that are being addressed. But it is nevertheless moving recognizably closer towards understanding what it is like to be a corvid, or for that matter, any nonhuman animal species. Concerning future research, we are hopeful that a close study of the life histories of corvids in the wild, as outlined Veit's recent pathological/life-history complexity framework (Veit 2023), will allow us to (i) come up with new experiments and (ii) better synthesize these experimental results into coherent experiental profiles for different corvid species.

## Data Availability

No datasets were generated or analysed during the current study.

## References

[CR1] Adriaense JE, Martin JS, Schiestl M, Lamm C, Bugnyar T (2019) Negative emotional contagion and cognitive bias in common ravens (*Corvus corax*). Proc Natl Acad Sci USA 116:11547–11552 31110007 10.1073/pnas.1817066116 PMC6561263

[CR3] Allen C, Trestman M (2017) Animal consciousness. In: Zalta EN (ed) The Stanford encyclopedia of philosophy (Winter 2017 ed). Stanford University, Metaphysics Research Lab

[CR4] Andrews K, Birch J, Sebo J, Sims T (2024) Background to the New York Declaration on Animal Consciousness. nydeclaration.com

[CR5] Baars BJ (2002) The conscious access hypothesis: origins and recent evidence. Trends Cogn Sci 6:47–52 11849615 10.1016/s1364-6613(00)01819-2

[CR6] Baciadonna L, Cornero F, Emery NJ, Clayton NS (2020) Convergent evolution of complex cognition: insights from the field of avian cognition into the study of self-awareness. Learn Behav 49:9–22 10.3758/s13420-020-00434-5 32661811

[CR7] Balduzzi D, Tononi G (2008) Integrated information in discrete dynamical systems: motivation and theoretical framework. PLoS Comput Biol 4:e1000091. 10.1371/journal.pcbi.1000091 18551165 10.1371/journal.pcbi.1000091 PMC2386970

[CR8] Balduzzi D, Tononi G (2009) Qualia: the geometry of integrated information. PLoS Comput Biol 5:e1000462. 10.1371/journal.pcbi.1000462 19680424 10.1371/journal.pcbi.1000462 PMC2713405

[CR9] Bang BG (1971) Functional anatomy of the olfactory system in 23 orders of birds. Acta Anat (Basel) 79:1–76 5133493 10.1159/isbn.978-3-318-01866-0

[CR10] Bebus SE, Small TW, Jones BC, Elderbrock EK, Schoech SJ (2016) Associative learning is inversely related to reversal learning and varies with nestling corticosterone exposure. Anim Behav 111:251–260

[CR11] Beecher MD, Brenowitz EA (2005) Functional aspects of song learning in songbirds. Trends Ecol Evol 20:143–149 16701358 10.1016/j.tree.2005.01.004

[CR12] Bingman VP, Gagliardo A, Hough GE, Ioalé P, Kahn MC, Siegel JJ (2005) The avian hippocampus, homing in pigeons and the memory representation of large-scale space. Integr Comp Biol 45:555–564 21676801 10.1093/icb/45.3.555

[CR13] Birch J (2017) Animal sentience and the precautionary principle. Anim Sent 2:1

[CR14] Birch J (2024) The Edge of Sentience: risk and precaution in humans, other animals, and AI. Oxford University Press

[CR15] Birch J, Schnell AK, Clayton NS (2020) Dimensions of animal consciousness. Trends Cogn Sci 24:789–801 32830051 10.1016/j.tics.2020.07.007 PMC7116194

[CR16] Blumberg MS, Wasserman EA (1995) Animal mind and the argument from design. Am Psychol 50:133–144 7726467 10.1037//0003-066x.50.3.133

[CR17] Bobrowicz K, Osvath M (2019) Cognition in the fast lane: ravens’ gazes are half as short as humans’ when choosing objects. Anim Behav Cognit 6:81–97

[CR18] Boeckle M, Schiestl M, Frohnwieser A, Gruber R, Miller R, Suddendorf T, Gray RD, Taylor AH, Clayton NS (2020) New Caledonian crows plan for specific future tool use. Proc R Soc B Biol Sci 287:20201490 10.1098/rspb.2020.1490 PMC7735258 33143583

[CR19] Bonadonna F, Mardon J (2013) Besides colours and songs, odour is the new black of avian communication. In: East ML, Dehnhard M (eds) Chemical signals in vertebrates, vol 12. Springer, pp 325–339

[CR20] Bond AB, Kamil A, Balda RP (2007) Serial reversal learning and the evolution of behavioral flexibility in three species of North American corvids (Gymnorhinus cyanocephalus, Nucifraga columbiana, Aphelocoma californica). J Comp Psychol 121:372–379 18085920 10.1037/0735-7036.121.4.372

[CR21] Boström JE, Dimitrova M, Canton C, Håstad O, Qvarnström A, Odeen A (2016) Ultra-rapid vision in birds. PLoS ONE 11:3–9 10.1371/journal.pone.0151099 PMC4798572 26990087

[CR23] Bresgunova O (2016) Cooperative breeding in corvids (Passeriformes, Corvidae). Biol Bull 43:693–706

[CR24] Brown MJ, Jones DN (2016) Cautious crows: neophobia in Torresian crows (*Corvus orru*) compared with three other corvids in suburban Australia. Ethology 122:726–733

[CR25] Browning H (2022) How should we study animal consciousness scientifically? J Conscious Stud 29:12–14

[CR26] Browning H (2020) If I could talk to the animals: measuring subjective animal welfare [dissertation]. Australian National University. 10.25911/5f1572fb1b5be

[CR27] Brüne M, Brüne-Cohrs U (2006) Theory of mind—evolution, ontogeny, brain mechanisms and psychopathology. Neurosci Biobehav Rev 30:437–455 16239031 10.1016/j.neubiorev.2005.08.001

[CR28] Buitron D, Nuechterlein GL (1985) Experiments on olfactory detection of food caches by black-billed magpies. The Condor 87:92–95

[CR29] Butler SR, Templeton JJ, Fernández‐Juricic E (2018) How do birds look at their world? a novel avian visual fixation strategy. Behav Ecol Sociobiol 72:1–11

[CR30] Byers BE, Kroodsma DE (2009) Female mate choice and songbird song repertoires. Anim Behav 77:13–22

[CR31] Call J (2007) Past and present challenges in theory of mind research in nonhuman primates. In: Progress in brain research, pp 341–353 10.1016/S0079-6123(07)64019-9 17920441

[CR32] Cheke LG, Clayton NS (2012) Eurasian jays (Garrulus glandarius) overcome their current desires to anticipate two distinct future needs and plan for them appropriately. Biol Let 8:171–175 22048890 10.1098/rsbl.2011.0909 PMC3297405

[CR33] Clark L, Avilova VK, Bean NJ (1993) Odor thresholds in passerines. Comp Biochem Physiol A Physiol 104:305–312

[CR34] Clark L, Hagelin J, Werner S (2014) The chemical senses in birds. In: C. Scanes (ed). Sturkie's Avian Physiology, Sixth edition. Academic Press, Elsevier Inc., Boston, MA, pp 89–111

[CR35] Clary D, Kelly DM (2011) Cache protection strategies of a non-social food-caching corvid, Clark’s nutcracker (Nucifraga columbiana). Anim Cogn 14:735–744 21538135 10.1007/s10071-011-0408-3

[CR36] Clary D, Kelly DM (2013) Are Clark’s nutcrackers (*Nucifraga columbiana*) able to discriminate knowledge states of human experimenters during an object-choice task? Evol Psychol 11:1–37 23864297 10.1177/147470491301100310 PMC10426872

[CR37] Clary D, Kelly DM (2016) Graded mirror self-recognition by Clark’s nutcrackers. Sci Rep 6:36459. 10.1038/srep36459 27811974 10.1038/srep36459 PMC5095562

[CR38] Clayton NS (2012) Corvid cognition: feathered apes. Nature 484:453–454

[CR39] Clayton NS, Dickinson A (1998) Episodic-like memory during cache recovery by scrub jays. Nature 395:272–274. 9751053 10.1038/26216

[CR40] Clayton NS, Emery NJ (2004) The mentality of crows: convergent evolution of intelligence in corvids and apes. Science 306(5703):1903–1907. 10.1126/science.1098410 15591194 10.1126/science.1098410

[CR41] Clayton NS, Emery NJ (2007a) The social life of corvids. Curr Biol 17:R652–R656 17714658 10.1016/j.cub.2007.05.070

[CR42] Clayton NS, Emery NJ (2007b) The social life of corvids. Curr Biol 17:16 10.1016/j.cub.2007.05.070 17714658

[CR43] Clayton NS, Emery NJ (2015) Avian models of human cognitive neuroscience: a proposal. Neuron 86:1330–1342 26087161 10.1016/j.neuron.2015.04.024

[CR44] Clayton NS, Krebs JR (1994) Memory for spatial and object-specific cues in food-storing and non-storing birds. J Comp Physiol Part A Neuroethol Sens Neural Behav Physiol 174:371–379

[CR45] Clayton NS, Yu KS, Dickinson A (2001a) Scrub jays (*Aphelocoma coerulescens*) form integrated memories of the multiple features of caching episodes. J Exp Psychol Anim Behav Process 27:17–29 11199511

[CR46] Clayton NS, Griffiths DP, Emery NJ, Dickinson A (2001b) Elements of episodic-like memory in animals. Philos Trans R Soc Lond Series B: Biol Sci 356(1413):1483–1491 10.1098/rstb.2001.0947 PMC1088530 11571038

[CR47] Clayton NS, Bussey TJ, Dickinson A (2003a) Can animals recall the past and plan for the future? Nat Rev Neurosci 4:685–691 12894243 10.1038/nrn1180

[CR48] Clayton NS, Yu KS, Dickinson A (2003b) Interacting cache memories: evidence for flexible memory use by Western scrub-jays (*Aphelocoma californica*). J Exp Psychol Anim Behav Process 29:14–22 12561130

[CR49] Cockburn A (2006) Prevalence of different modes of parental care in birds. Proc R Soc B Biol Sci 273:1375–1383 10.1098/rspb.2005.3458 PMC1560291 16777726

[CR50] Colombo M, Broadbent N (2000) Is the avian hippocampus a functional homologue of the mammalian hippocampus? Neurosci Biobehav Rev 24:465–484 10817844 10.1016/s0149-7634(00)00016-6

[CR51] Corballis MC (2014) Mental time travel: how the mind escapes from the present. Cosmology 18:139–145

[CR52] Crump A, Arnott G, Bethell EJ (2018) Affect-driven attention biases as animal welfare indicators: review and methods. Animals 8:136 30087230 10.3390/ani8080136 PMC6115853

[CR53] Crystal JD (2013) Remembering the past and planning for the future in rats. Behav Proc 93:39–49 10.1016/j.beproc.2012.11.014 PMC3582767 23219951

[CR54] Crystal JD (2021) Evaluating evidence from animal models of episodic memory. Journal of Experimental Psychology: Animal Learning and Cognition 47:337 34618532 10.1037/xan0000294

[CR55] Dainton B (2010) Temporal consciousness, The Stanford Encyclopedia of Philosophy (Fall 2024 Edition), Edward N. Zalta & Uri Nodelman (eds.). https://plato.stanford.edu/archives/fall2024/entries/consciousnesstemporal/

[CR56] Dally JM, Emery NJ, Clayton NS (2006) Food-caching western scrub-jays keep track of who was watching when. Science 312:1662–1665 16709747 10.1126/science.1126539

[CR57] Dally JM, Emery NJ, Clayton NS (2010) Avian theory of mind and counter-espionage by food-caching western scrub-jays (*Aphelocoma californica*). Eur J Dev Psychol 7:17–37

[CR58] Davidson G, Miller R, Loissel E, Cheke LG, Clayton NS (2017) The development of support intuitions and object causality in juvenile Eurasian jays (*Garrulus glandarius*). Sci Rep 7:1–11 28053306 10.1038/srep40062 PMC5215467

[CR59] Davies JR, Clayton NS (2024) Is episodic-like memory like episodic memory? Philos Trans R Soc Lond Part B Biol Sci 379:20230397 10.1098/rstb.2023.0397 PMC11449162 39278246

[CR60] Davies JR, Garcia-Pelegrin E, Baciadonna L, Pilenga C, Favaro L, Clayton NS (2022) Episodic-like memory in common bottlenose dolphins. Curr Biol 32:3436–3442 35882234 10.1016/j.cub.2022.06.032

[CR61] Davies JR, Garcia-Pelegrin E, Clayton NS (2024a) Eurasian jays (*Garrulus glandarius*) show episodic-like memory through the incidental encoding of information. PLoS ONE 19:e0301298 38748646 10.1371/journal.pone.0301298 PMC11095760

[CR62] Davies JR, Keuneke LS, Clayton NS, Davidson GL (2024b) Episodic-like memory in wild free-living blue tits and great tits. Curr Biol 34:3593–3602 38964317 10.1016/j.cub.2024.06.029

[CR63] Dawkins MS (1980) Animal suffering: the science of animal welfare. Chapman and Hall

[CR64] de Lafuente V, Romo R (2006) Neural correlate of subjective sensory experience gradually builds up across cortical areas. Proc Natl Acad Sci USA 103:14266–14271 16924098 10.1073/pnas.0605826103 PMC1551903

[CR65] de Haan EH, Corballis PM, Hillyard SA, Marzi CA, Seth A, Lamme VA, Volz L, Fabri M, Schechter E, Bayne T et al (2020) Split brain: what we know now and why this is important for understanding consciousness. Neuropsychol Rev 30:224–233 32399946 10.1007/s11065-020-09439-3 PMC7305066

[CR68] Drayton L, Santos L (2013) Capuchins’ (*Cebus apella*) sensitivity to others’ goal-directed actions in a helping context. Anim Cogn 17:689–700 24146217 10.1007/s10071-013-0700-5

[CR69] Dufour V, Wascher CAF, Braun A, Miller R, Bugnyar T (2012) Corvids can decide if a future exchange is worth waiting for. Biol Let 8:201–204 21920957 10.1098/rsbl.2011.0726 PMC3297375

[CR70] Dufour V, Broihanne M-H, Wascher CAF (2019) Corvids avoid odd evaluation by following simple rules in a risky exchange task. Ethology 00:1–12

[CR71] Dukas R, Kamil AC (2000) The cost of limited attention in blue jays. Behav Ecol 11:502–506

[CR72] Dukas R, Kamil AC (2001) Limited attention: the constraint underlying search image. Behav Ecol 12:192–199

[CR73] Duncan IJ (2002) Poultry welfare: science or subjectivity? Br Poult Sci 43:643–652 12555888 10.1080/0007166021000025109

[CR74] Edelman DB, Baars BJ, Seth AK (2005) Identifying hallmarks of consciousness in non-mammalian species. Conscious Cogn 14:169–187 15766896 10.1016/j.concog.2004.09.001

[CR75] Emery NJ (1998) Are corvids “feathered apes”? Cognitive evolution in crows, rooks and jackdaws. In: Watanabe S (ed) Comparative Analysis of Minds. Keio University Press, pp 181–213

[CR76] Emery NJ, Clayton NS (2001) Effects of experience and social context on prospective caching strategies by scrub jays. Nature 414:443–446 11719804 10.1038/35106560

[CR240] Emery NJ, Clayton NS (2004) The Mentality of Crows: Convergent Evolution of Intelligence in Corvids and Apes. Science 306:(5703):1903–1907. 10.1126/science.1098410 15591194 10.1126/science.1098410

[CR79] Emery NJ, Clayton NS (2009) Comparative social cognition. Annu Rev Psychol 60:87–113 18831684 10.1146/annurev.psych.60.110707.163526

[CR80] Emery NJ, Clayton NS (2015) Do birds have the capacity for fun? Curr Biol 25:R16 25562293 10.1016/j.cub.2014.09.020

[CR81] Fraser O, Bugnyar T (2010a) The quality of social relationships in ravens. Anim Behav 79:927–933 25821236 10.1016/j.anbehav.2010.01.008 PMC4373546

[CR82] Fraser ON, Bugnyar T (2010b) Do ravens show consolation? responses to distressed others. PLoS ONE 5:e10605 20485685 10.1371/journal.pone.0010605 PMC2868892

[CR83] Gallup GG (1970) Chimpanzees: self-recognition. Science 167:86–87 4982211 10.1126/science.167.3914.86

[CR84] Godfrey-Smith P (2021) Integration, lateralization, and animal experience. Mind Lang 36:285–296

[CR85] Gossette RL, Gossette MF, Riddell W (1966) Comparisons of successive discrimination reversal performances among closely and remotely related avian species. Anim Behav 14:560–564 5972814 10.1016/s0003-3472(66)80060-x

[CR86] Goursot C, Düpjan S, Puppe B, Leliveld LM (2021) Affective styles and emotional lateralization: a promising framework for animal welfare research. Appl Anim Behav Sci 237:105279

[CR87] Greggor AL, Clayton NS, Fulford AJC, Thornton A (2016) Street smart: faster approach towards litter in urban areas by highly neophobic corvids and less fearful birds. Anim Behav 117:123–133 27429456 10.1016/j.anbehav.2016.03.029 PMC4938798

[CR88] Greggor AL, Masuda B, Flanagan AM, Swaisgood RR (2020) Age-related patterns of neophobia in an endangered island crow: implications for conservation and natural history. Anim Behav 160:61–68

[CR90] Grodzinski U, Clayton NS (2010) Problems faced by food-caching corvids and the evolution of cognitive solutions. Philos Trans R Soc Lond Part B Biol Sci 365:977–987 10.1098/rstb.2009.0210 PMC2830244 20156820

[CR91] Güntürkün O (2005) The avian “prefrontal cortex” and cognition. Curr Opin Neurobiol 15:686–693 16263260 10.1016/j.conb.2005.10.003

[CR92] Güntürkün O, Ströckens F, Scarf D, Colombo M (2017) Apes, feathered apes, and pigeons: differences and similarities. Curr Opin Behav Sci 16:35–40

[CR222] Hahner L, Nieder A (2025) Volitional spatial attention is lateralized in crows. Proc R Soc B Biol Sci 292(2039). 10.1098/rspb.2024.2540 10.1098/rspb.2024.2540 PMC11775601 39876722

[CR93] Halley DJ (2001) Interspecific dominance and risk-taking in three species of corvid scavenger. J Yamashina Inst Ornithol 33:44–50

[CR94] Hare B, Call J, Tomasello M (2001) Do chimpanzees know what conspecifics know? Anim Behav 61:139–151 11170704 10.1006/anbe.2000.1518

[CR95] Harriman AE, Berger RH (1986) Olfactory acuity in the common raven (*Corvus corax*). Physiol Behav 36:257–262 3960998 10.1016/0031-9384(86)90013-2

[CR96] Hassabis D, Kumaran D, Vann SD, Maguire EA (2007) Patients with hippocampal amnesia cannot imagine new experiences. Proc Natl Acad Sci USA 104:1726–1731 17229836 10.1073/pnas.0610561104 PMC1773058

[CR97] Hill CS (2018) Unity of consciousness. Wiley Interdiscipl Rev Cognit Sci 9:e1465 10.1002/wcs.1465 29809308

[CR98] Hillemacher S et al (2023) Roosters do not warn the bird in the mirror: the cognitive ecology of mirror self-recognition. PLoS ONE 18:e0291416. 10.1371/journal.pone.0291416 37878556 10.1371/journal.pone.0291416 PMC10599514

[CR99] Hilleman F, Bugnyar T, Kotrschal K, Wascher CAF (2014) Waiting for better, not for more: corvids respond to quality in two delay maintenance tasks. Anim Behav 90:1–10 25892738 10.1016/j.anbehav.2014.01.007 PMC4398876

[CR100] Holland SM, Smulders TV (2011) Do humans use episodic memory to solve a what-where-when memory task? Anim Cogn 14:95–102 20661614 10.1007/s10071-010-0346-5

[CR101] Humphrey NK (1976) The social function of intellect. In: Bateson P, Hinde R (eds) Growing points in ethology. Cambridge University Press, Cambridge, pp 303–317

[CR102] Hunt GR (1996) Manufacture and use of hook-tools by New Caledonian crows. Nature 379:249–251

[CR241] Jacobs IF, Osvath M, Osvath H, Mioduszewska B, von Bayern AM, Kacelnik A (2014) Object caching in corvids: incidence and significance. Behavioural processes 102:25–32 24333834 10.1016/j.beproc.2013.12.003

[CR103] James W, Burkhardt F, Bowers F, Skrupskelis IK (1890) The principles of psychology, vol 1. Macmillan, London

[CR104] Janik VM, Slater PJB (2000) The different roles of social learning in vocal communication. Anim Behav 60:1–11 10924198 10.1006/anbe.2000.1410

[CR105] Jolly A (1966) Lemur social behavior and primate intelligence. Science 153:501–506 5938775 10.1126/science.153.3735.501

[CR106] Jones MP, Pierce KE Jr, Ward D (2007) Avian vision: a review of form and function with special consideration to birds of prey. J Exotic Pet Med 16:69–87

[CR107] Kabadayi C, Taylor LA, von Bayern AM, Osvath M (2016) Ravens, New Caledonian crows and jackdaws parallel great apes in motor self-regulation despite smaller brains. R Soc Open Sci 3:160104 27152224 10.1098/rsos.160104 PMC4852647

[CR108] Kaunitz LN, Rowe EG, Tsuchiya N (2016) Large capacity of conscious access for incidental memories in natural scenes. Psychol Sci 27:1266–1277 27507869 10.1177/0956797616658869

[CR109] Kenward B, Schloegl C, Rutz C, Weir AA, Bugnyar T, Kacelnik A (2011) On the evolutionary and ontogenetic origins of tool-oriented behaviour in New Caledonian crows (Corvus moneduloides). Biol J Lin Soc 102:870–877 10.1111/j.1095-8312.2011.01613.x PMC4398869 25892825

[CR110] Klein SB, Loftus J, Kihlstrom JF (2002) Memory and temporal experience: the effects of episodic memory loss on an amnesic patient’s ability to remember the past and imagine the future. Soc Cogn 20:353–379

[CR111] Koboroff A, Kaplan G, Rogers LJ (2008) Hemispheric specialization in Australian magpies (Gymnorhina tibicen) shown as eye preferences during response to a predator. Brain Res Bull 76:304–306 18498946 10.1016/j.brainresbull.2008.02.015

[CR112] Koch C, Tononi G (2011) A test for consciousness. [Details not provided] 10.1038/scientificamerican0611-44 21608402

[CR113] Kolers PA, von Grünau M (1976) Shape and color in apparent motion. Vision Res 16:329–335 941407 10.1016/0042-6989(76)90192-9

[CR114] Krupenye C, Call J (2019) Theory of mind in animals: current and future directions. Wires Cognit Sci 10:6 10.1002/wcs.1503 31099977

[CR115] Lagisz M, Zidar J, Nakagawa S, Neville V, Sorato E, Paul ES, Bateson M, Mendl M, Løvlie H (2020) Optimism, pessimism and judgement bias in animals: a systematic review and meta-analysis. Neurosci Biobehav Rev 118:3–17 32682742 10.1016/j.neubiorev.2020.07.012

[CR117] Landis C (1954) Determinants of the critical flicker-fusion threshold. Physiol Rev 34:259–286 13155188 10.1152/physrev.1954.34.2.259

[CR119] Low P, Panksepp J, Reiss D, Edelman D, van Swinderen B, Koch C (2012) The Cambridge Declaration on Consciousness. [Conference proceedings, July, Cambridge, England]

[CR120] Maginnity M, Grace R (2014) Visual perspective taking by dogs (Canis familiaris) in a Guesser-Knower task: evidence for a canine theory of mind? Anim Cogn 17:1375–1392 24950722 10.1007/s10071-014-0773-9

[CR121] Manns J, Eichenbaum H (2007) Evolution of the hippocampus. In: Elsevier eBooks, pp 465–489

[CR122] Martin GR (2007) Visual fields and their functions in birds. J Ornithol 148:547–562

[CR123] Martin GR (2009) What is binocular vision for? a birds’ eye view. J vis 9:14 20053077 10.1167/9.11.14

[CR124] Martin GR (2014) The subtlety of simple eyes: the tuning of visual fields to perceptual challenges in birds. Philos Trans R Soc Lond Part B Biol Sci 369:20130040 10.1098/rstb.2013.0040 PMC3886328 24395967

[CR125] Martin RJ, Martin GK, Roberts WA, Sherry DF (2021) No evidence for future planning in Canada jays (*Perisoreus canadensis*). Biol Let 17:20210504 34875182 10.1098/rsbl.2021.0504 PMC8651407

[CR126] Matthews J, Schröder P, Kaunitz L, Van Boxtel JJ, Tsuchiya N (2018) Conscious access in the near absence of attention: critical extensions on the dual-task paradigm. Philos Trans R Soc Lond Part B Biol Sci 373:20170352 10.1098/rstb.2017.0352 PMC6074075 30061465

[CR127] Matthews J, Wu J, Corneille V, Hohwy J, van Boxtel J, Tsuchiya N (2019) Sustained conscious access to incidental memories in RSVP. Atten Percept Psychophys 81:188–204 30291551 10.3758/s13414-018-1600-1

[CR128] McCoy DE, Schiestl M, Neilands P, Hassall R, Gray RD, Taylor AH (2019) New Caledonian crows behave optimistically after using tools. Curr Biol 29:2737-2742.e3 31378612 10.1016/j.cub.2019.06.080

[CR129] Meisami E, Bhatnagar KP (1998) Structure and diversity in mammalian accessory olfactory bulb. Microsc Res Tech 43:476–499 9880163 10.1002/(SICI)1097-0029(19981215)43:6<476::AID-JEMT2>3.0.CO;2-V

[CR130] Mellor DJ, Beausoleil NJ, Littlewood KE, McLean AN, McGreevy PD, Jones B, Wilkins C (2020) The 2020 Five Domains model: including human–animal interactions in assessments of animal welfare. Animals 10:1870 33066335 10.3390/ani10101870 PMC7602120

[CR131] Mendl M, Burman OH, Parker RM, Paul ES (2009) Cognitive bias as an indicator of animal emotion and welfare: emerging evidence and underlying mechanisms. Appl Anim Behav Sci 118:161–181

[CR132] Miller R, Lambert ML, Frohnwieser A, Brecht KF, Bugnyar T, Crampton I, Garcia-Pelegrin E, Gould K, Greggor AL, Izawa E-I, Kelly DM, Li Z, Luo Y, Luong LB, Massen JJ, Nieder A, Reber SA, Schiestl M, Seguchi A, Sepehri P, Stevens JR, Taylor AH, Wang L, Wolff LM, Zhang Y, Clayton NS (2022) Socio-ecological correlates of neophobia in corvids. Curr Biol 32:74–85 34793696 10.1016/j.cub.2021.10.045

[CR133] Molina-Morales M, Castro J, Albaladejo G, Parejo D (2020) Precise cache detection by olfaction in a scatter-hoarder bird. Anim Behav 167:185–191

[CR134] Moreno-Rueda G (2017) Preen oil and bird fitness: a critical review of the evidence. Biol Rev 92:2131–2143 28231637 10.1111/brv.12324

[CR135] Morris RGM, Frey U (1997) Hippocampal synaptic plasticity: role in spatial learning or the automatic recording of attended experience? Philos Trans R Soc Lond Part B Biol Sci 352:1489–1503 10.1098/rstb.1997.0136 PMC1692060 9368938

[CR136] Moulton DG (1967) Olfaction in mammals. Am Zool 7:421–429 6077376 10.1093/icb/7.3.421

[CR137] Nagel T (1974) What is it like to be a bat? Philos Rev 83:435

[CR138] Nieder A, Wagener L, Rinnert P (2020) A neural correlate of sensory consciousness in a corvid bird. Science 369:1626–1629 32973028 10.1126/science.abb1447

[CR139] Ortega LJ, Stoppa K, Güntürkün O, Troje NF (2008) Limits of intraocular and interocular transfer in pigeons. Behav Brain Res 193:69–78 18547658 10.1016/j.bbr.2008.04.022

[CR140] Ostojíc L, Shaw RC, Cheke LG, Clayton NS (2013) Evidence suggesting that desire-state attribution may govern food sharing in Eurasian jays. Proc Natl Acad Sci USA 110:4123–4128 23382187 10.1073/pnas.1209926110 PMC3593841

[CR141] Osvath M, Sima M (2014) Sub-adult ravens synchronize their play: a case of emotional contagion. Anim Behav Cognit 1:197–205

[CR142] Panoz-Brown D, Iyer V, Carey LM, Sluka CM, Rajic G, Kestenman J, Crystal JD (2018) Replay of episodic memories in the rat. Curr Biol 28:1628–1634 29754898 10.1016/j.cub.2018.04.006 PMC5964044

[CR143] Penn DC, Povinelli DJ (2007) On the lack of evidence that nonhuman animals possess anything remotely resembling a “theory of mind.” Philos Trans R Soc Lond Part B Biol Sci 362:731–744 10.1098/rstb.2006.2023 PMC2346530 17264056

[CR144] Pinto Y, de Haan EH, Lamme VA (2017) The split-brain phenomenon revisited: a single conscious agent with split perception. Trends Cogn Sci 21:835–851 28958646 10.1016/j.tics.2017.09.003

[CR145] Povinelli DJ (1994) Comparative studies of animal mental state attribution: a reply to Heyes. Anim Behav 48:239–241

[CR146] Povinelli DJ, Rulf AB, Bierschwale DT (1994) Absence of knowledge attribution and self-recognition in young chimpanzees (*Pan troglodytes*). J Comp Psychol 108:74–80 8174347 10.1037/0735-7036.108.1.74

[CR147] Premack D, Woodruff G (1978) Does the chimpanzee have a theory of mind? Behav Brain Sci 1:515–526

[CR148] Prior H, Schwarz A, Güntürkün O (2008) Mirror-induced behavior in the magpie (*Pica pica*): evidence of self-recognition. PLoS Biol 6:8 10.1371/journal.pbio.0060202 PMC2517622 18715117

[CR149] Qianchen L, Gallagher RM, Tsuchiya N (2022) How much can we differentiate at a brief glance: revealing the truer limit in conscious contents through the Massive Report Paradigm (MRP). R Soc Open Sci 9:210394 35619998 10.1098/rsos.210394 PMC9128849

[CR150] Raby CR, Alexis DM, Dickinson A, Clayton NS (2007) Planning for the future by western scrub-jays. Nature 445:919–921 17314979 10.1038/nature05575

[CR151] Rattenborg NC, Martinez-Gonzalez D (2011) A bird-brain view of episodic memory. Behav Brain Res 222:236–245 21420438 10.1016/j.bbr.2011.03.030

[CR152] Reznikova ZI (2007) Animal intelligence: from individual to social cognition. Cambridge University Press, Cambridge

[CR153] Riters LV (2011) Pleasure seeking and birdsong. Neurosci Biobehav Rev 35:1837–1845 21251924 10.1016/j.neubiorev.2010.12.017 PMC3091979

[CR154] Roberts WA, Feeney MC, MacPherson K, Petter M, McMillan N, Musolino E (2008) Episodic-like memory in rats: is it based on when or how long ago? Science 320:113–115 18388296 10.1126/science.1152709

[CR155] Rogers LJ (2008) Development and function of lateralization in the avian brain. Brain Res Bull 76:235–244 18498936 10.1016/j.brainresbull.2008.02.001

[CR156] Rogers L, Kaplan G (2006) An eye for a predator: lateralization in birds, with particular reference to behavioural and morphological asymmetries in vertebrates. In: Yegor M, Wallace Deckel A (eds) Behavioural and morphological asymmetries in vertebrates. Landes Bioscience, p 47

[CR157] Rosenbaum RS, Köhler S, Schacter DL, Moscovitch M, Westmacott R, Black SE, Gao F, Tulving E (2005) The case of KC: contributions of a memory-impaired person to memory theory. Neuropsychologia 43:989–1021 15769487 10.1016/j.neuropsychologia.2004.10.007

[CR158] Rubene D (2009) Functional differences in avian colour vision: a behavioural test of critical flicker fusion frequency (CFF) for different wavelengths and light intensities. Uppsala University

[CR159] Rutz C, Hunt GR, St Clair JJH (2018) Corvid technologies: how do New Caledonian crows get their tool designs? Curr Biol 28:R1109–R1111 30253153 10.1016/j.cub.2018.08.031

[CR160] Seed A, Clayton NS, Emery NJ (2008) Cooperative problem solving in rooks (*Corvus frugilegus*). Proc R Soc B Biol Sci 275:1421–1429 10.1098/rspb.2008.0111 PMC2602707 18364318

[CR161] Shankar H, Pesudovs K (2007) Critical flicker fusion test of potential vision. J Cataract Refract Surg 33:232–239 17276263 10.1016/j.jcrs.2006.10.042

[CR162] Shaw RC, Clayton NS (2014) Pilfering Eurasian jays use visual and acoustic information to locate caches. Anim Cogn 17:1281–1288 24889656 10.1007/s10071-014-0763-y

[CR163] Sheridan CL, Lang S, Knappenberger M, Albers C, Loper R, Tillett B, Crystal JD (2024) Replay of incidentally encoded episodic memories in the rat. Curr Biol 34:641–647 38218186 10.1016/j.cub.2023.12.043

[CR164] Sherry DF (2006) Neuroecology. Annu Rev Psychol 57:167–197 16318593 10.1146/annurev.psych.56.091103.070324

[CR165] Siegel R (1970) Apparent movement detection in the pigeon 1. J Exp Anal Behav 14:93–97 4194436 10.1901/jeab.1970.14-93 PMC1333704

[CR166] Siegel R (1971) Apparent movement and real movement detection in the pigeon: stimulus generalization. J Exp Anal Behav 16:189–192 5166169 10.1901/jeab.1971.16-189 PMC1333866

[CR167] Simons JS, Spiers HJ (2003) Prefrontal and medial temporal lobe interactions in long-term memory. Nat Rev Neurosci 4:637–648 12894239 10.1038/nrn1178

[CR169] Soler M, Pérez-Contreras T, Peralta-Sánchez JM (2014) Mirror-mark tests performed on jackdaws reveal potential methodological problems in the use of stickers in avian mark-test studies. PLoS ONE 9:e86193 24475085 10.1371/journal.pone.0086193 PMC3903501

[CR171] Špinka M (2019) Animal agency, animal awareness and animal welfare. Anim Welf 28:11–20

[CR172] Suddendorf T, Corballis MC (1997) Mental time travel and the evolution of the human mind. Genet Soc Gen Psychol Monogr 123:133–167 9204544

[CR173] Suddendorf T, Corballis MC (2010) Behavioural evidence for mental time travel in nonhuman animals. Behav Brain Res 215:292–298 19962409 10.1016/j.bbr.2009.11.044

[CR174] Suddendorf T, Addis DR, Corballis MC (2009) Mental time travel and the shaping of the human mind. Philos Trans R Soc Lond Part B Biol Sci 364:1317–1324 10.1098/rstb.2008.0301 PMC2666704 19528013

[CR175] Supèr H, Spekreijse H, Lamme VAF (2001) Two distinct modes of sensory processing observed in monkey primary visual cortex (V1). Nat Neurosci 4:304–310 11224548 10.1038/85170

[CR176] Swift K, Marzluff JM (2015) Wild American crows gather around their dead to learn about danger. Anim Behav 109:187–197

[CR177] Swift K, Marzluff JM (2018) Occurrence and variability of tactile interactions between wild American crows and dead conspecifics. Philos Trans R Soc Lond Part B Biol Sci 373:20170259 10.1098/rstb.2017.0259 PMC6053988 30012745

[CR178] Taniguchi K, Saito S, Taniguchi K (2011) Phylogenic outline of the olfactory system in vertebrates. J Vet Med Sci 73(2):139–147 20877153 10.1292/jvms.10-0316

[CR180] Teschke I, Wascher CAF, Scriba MF, von Bayern AMP, Huml V, Siemers B, Tebbich S (2013) Did tool-use evolve with enhanced physical cognitive abilities? Philos Trans R Soc Lond Part B Biol Sci 368:20120418 10.1098/rstb.2012.0418 PMC4027416 24101628

[CR181] Tinbergen N (1963) On aims and methods of ethology. Z Tierpsychol 20:410–433

[CR182] Tononi G (2004) An information integration theory of consciousness. BMC Neurosci 5:42 15522121 10.1186/1471-2202-5-42 PMC543470

[CR183] Tononi G (2005) Consciousness, information integration, and the brain. Prog Brain Res 150:109–126 16186019 10.1016/S0079-6123(05)50009-8

[CR184] Tononi G (2008) Consciousness as integrated information: a provisional manifesto. Biol Bull 215:216–242 19098144 10.2307/25470707

[CR185] Tononi G (2010) Information integration: its relevance to brain function and consciousness. Arch Ital Biol 148:299–322 21175016

[CR186] Tononi G (2012a) The integrated information theory of consciousness: an updated account. Arch Ital Biol 150:56–90 23165867 10.4449/aib.v149i5.1388

[CR187] Tononi G (2012b) Phi: a voyage from the brain to the soul. Pantheon Books, New York

[CR188] Tononi G (2015) Integrated information theory. Scholarpedia 10:4164

[CR189] Tononi G, Boly M, Massimini M, Koch C (2016) Integrated information theory: from consciousness to its physical substrate. Nat Rev Neurosci 17:450–461 27225071 10.1038/nrn.2016.44

[CR190] Tononi G, Koch C (2008) The neural correlates of consciousness—an update. [Details not provided] 10.1196/annals.1440.004 18400934

[CR191] Troscianko J, von Bayern AMP, Chappell J, Rutz C, Martin GR (2012) Extreme binocular vision and a straight bill facilitate tool use in New Caledonian crows. Nat Commun 3:1–7 10.1038/ncomms2111 23047668

[CR192] Tulving E (1972) Episodic and semantic memory. Academic Press, New York

[CR193] Tulving E (1983) Elements of episodic memory. Oxford University Press, Oxford

[CR194] Tulving E (1985) Memory and consciousness. Can Psychol 26:1

[CR195] Tulving E (2005) Episodic memory and autonoesis: uniquely human? In: Terrace HS, Metcalfe J (eds) The missing link in cognition: origins of self-reflective consciousness. Oxford University Press, pp 3–56

[CR196] Tulving E, Markowitsch HJ (1998) Episodic and declarative memory: role of the hippocampus. Hippocampus 8:198–204 9662134 10.1002/(SICI)1098-1063(1998)8:3<198::AID-HIPO2>3.0.CO;2-G

[CR197] Vallortigara G (2000) Comparative neuropsychology of the dual brain: a stroll through animals’ left and right perceptual worlds. Brain Lang 73:189–219 10856174 10.1006/brln.2000.2303

[CR198] Van Horik J, Clayton NS, Emery NJ (2012) Convergent evolution of cognition in corvids, apes and other animals. In: Schackelford T, Vonk J (eds) The Oxford handbook of comparative evolutionary psychology, chapter 5. Oxford University Press, pp 80–101

[CR199] Vander Wall S (2003) Reciprocal pilferage and the evolution of food-hoarding behavior. Behav Ecol 14:656–667

[CR200] Veit W (2023) A philosophy for the science of animal consciousness. Routledge, New York

[CR201] Veit W (2021) Health, agency, and the evolution of consciousness [dissertation]. University of Sydney

[CR202] Veit W (2022) Complexity and the evolution of consciousness. Biol Theory 18:175–190

[CR203] Vernouillet A, Casidsid H, Kelly DM (2021) Pinyon jays (*Gymnorhinus cyanocephalus*) do not discriminate between pilfering and non-pilfering conspecifics. Learn Behav 49:23–35. 10.3758/s13420-020-00450-5 33269437 10.3758/s13420-020-00450-5

[CR204] Vernouillet A, Clary D, Kelly DM (2023) Social information used to elicit cache protection responses differs between pinyon jays and Clark’s nutcrackers. Behav Ecol Sociobiol 77:50–65

[CR205] Volz LJ, Gazzaniga MS (2017) Interaction in isolation: 50 years of insights from split-brain research. Brain 140:2051–2060 29177496 10.1093/brain/awx139

[CR206] von Bayern A, Emery NJ (2009) Jackdaws respond to human attentional states and communicative cues in different contexts. Curr Biol 19:602–606 19345101 10.1016/j.cub.2009.02.062

[CR207] Vonk J (2019) Emotional contagion or sensitivity to behavior in ravens? Proc Natl Acad Sci USA 116:18168 31431538 10.1073/pnas.1909864116 PMC6744878

[CR208] Wagener L, Nieder A (2020) Categorical auditory working memory in crows. iScience 23:101737 33225245 10.1016/j.isci.2020.101737 PMC7662871

[CR210] Wascher CAF, Bugnyar T (2013) Behavioral responses to inequity in reward distribution and working effort in crows and ravens. PLoS ONE 8:e56885 23437262 10.1371/journal.pone.0056885 PMC3577644

[CR211] Wascher CAF, Dufour V, Bugnyar T (2012) Carrion crows cannot overcome impulsive choice in a quantitative exchange task. Front Psychol 3:1–6 22529833 10.3389/fpsyg.2012.00118 PMC3328082

[CR212] Wascher CAF, Hillemann F, Canestrari D, Baglione V (2015) Carrion crows learn to discriminate between calls of reliable and unreliable conspecifics. Anim Cogn 18:1181–1185 26067282 10.1007/s10071-015-0879-8

[CR213] Wascher CAF, Feider B, Bugnyar T, Dufour V (2019) Crows and common ravens do not reciprocally exchange tokens with a conspecific to gain food rewards. Ethology 00:1–10

[CR214] Wascher CAF, Allen K, Szipl G (2021) Learning and motor inhibitory control in crows and domestic chickens. R Soc Open Sci 8:210504 34703616 10.1098/rsos.210504 PMC8527213

[CR215] Wearing D (2005) Forever today: a memoir of love and amnesia. Random House

[CR216] Weir AA, Kenward B, Chappell J, Kacelnik A (2004) Lateralization of tool use in New Caledonian crows (*Corvus moneduloides*). Proc R Soc Lond Part B Biol Sci 271(suppl 5):S344–S346 10.1098/rsbl.2004.0183 PMC1810068 15504013

[CR217] West MJ, King AP (1996) Social learning: Synergy and songbirds. In C. M. Heyes & B. G. Galef, Jr. (Eds.), Social learning in animals: The roots of culture (pp. 155–178). Academic Press.

[CR219] Zentall TR, Clement TS, Bhatt RS, Allen J (2001) Episodic-like memory in pigeons. Psychon Bull Rev 8:685–690 11848586 10.3758/bf03196204

[CR220] Zhou W, Crystal JD (2009) Evidence for remembering when events occurred in a rodent model of episodic memory. Proc Natl Acad Sci USA 106:9525–9529 19458264 10.1073/pnas.0904360106 PMC2695044

[CR221] Zinkivskay A, Nazir F, Smulders TV (2009) What-where-when memory in magpies (*Pica pica*). Anim Cogn 12:119–125 18670793 10.1007/s10071-008-0176-x

